# A replicated, whole-insect transcriptomic dataset for *Nezara viridula* (southern green stink bug) spanning developmental stadia and sexes

**DOI:** 10.1016/j.dib.2024.111106

**Published:** 2024-11-04

**Authors:** Michael E. Sparks, Daniel Kuhar, Donald C. Weber, Dawn E. Gundersen-Rindal

**Affiliations:** USDA-ARS Invasive Insect Biocontrol and Behavior Laboratory, Beltsville, MD 20705, United States

**Keywords:** Transcriptomics, Gene expression, Southern green stink bug, Invasive insects, Agricultural pests

## Abstract

The southern green stink bug, *Nezara viridula* (Hemiptera: Pentatomidae), is a serious agricultural pest insect causing economic damage in the western hemisphere. Although transcriptome resources exist for adults of this species at both midgut-only and whole-insect levels, data for juvenile developmental stadia are not currently available. Sequence data reported in this study close this gap, providing a substantial increase of extrinsic evidence for use in improving gene annotations in the southern green stink bug genome project, for example. Analysis of these data identify 21,380 putative transcripts associated with differentially expressed genes, providing a list of potential targets for downstream experimental characterization of gene function.

Specifications Table

**Table:** 

Subject	Genetics, Genomics and Biological Sciences.
Specific subject area	Whole-insect transcriptomics spanning multiple developmental stadia of the southern green stink bug (*Nezara viridula*), an agricultural nuisance insect species.
Type of data	Illumina PE150 RNA-Seq data, reads trimmed per quality information (FASTQ format).
Data collection	Three whole-insect biological replicates apiece were prepared for each of 2nd and 4th nymphal instars, and approximately seven-day-old unmated male and female adults. *N. viridula* insects were reared in a culture maintained at the Beltsville Agricultural Research Center in Beltsville, Maryland, USA. Ten individuals were pooled per 2nd instar replicate, and five individuals for all others. Libraries were prepared and sequenced on an Illumina HiSeq 2000 instrument at the University of Georgia Genomics Facility (Athens, Georgia, USA); this vendor also performed quality-based read trimming on resulting sequences.
Data source location	Biological sequence data are stored and made publicly available at the NCBI in Bethesda, Maryland, USA.
Data accessibility	Repository name: National Library of Medicine - National Center for Biotechnology Information – BioProject Division Data identification number: BioProject accession number PRJNA1151593 Direct URL to data: https://www.ncbi.nlm.nih.gov/bioproject/?term=PRJNA1151593
Related research article	*None*

## 1 Value of the Data

• These data expand upon the developmental stadia already represented in the literature and public sequence databases. Until now, transcriptomic data have not been available characterizing juvenile life stages of *N. viridula*. This is a critical deficiency in that certain genes may only be expressed in juveniles and are thus not represented among previously released datasets. Identifying nymph-specific genes is valuable, in that these may constitute interesting experimental targets for gene function characterization. Repressing such genes in applied settings may disrupt the insect's life cycle, thereby providing a means for effective population control.
• These data also expand on tissue types available: certain other currently available *N. viridula* transcriptomic datasets only characterize the host at the midgut level, and thus may potentially overlook important transcripts specifically expressed in other, non-midgut tissue types.
• Owing to use of an identical, statistically replicated experimental design intended to minimize any distorting influence of confounding variables (e.g., identical library construction protocols were followed and libraries were processed on a single sequencing instrument), comparison of samples within this dataset for differences in gene expression is more statistically reliable than would be possible when comparing these data with those generated by earlier transcriptomics experiments. A reliable listing of such differentially expressed genes can, for instance, be used by functional genomics researchers: such a list provides compelling targets for downstream reverse genetics experiments whose objective is to better understand basic insect biology.
• These data could be used as a form of extrinsic homology information that can help to improve the accuracy of gene finding efforts in southern green stink bug whole-genome sequencing projects such as that which has produced the high quality, currently unpublished assembly available at GenBank under accession number GCA_928085145.1.

## 2 Background

*Nezara viridula* (Hemiptera: Pentatomidae), the southern green stink bug, is an agricultural pest insect feeding on more than 30 families of plants and causing substantial economic damage in tropical, subtropical and warm-temperate regions, including in South America and southern regions of the contiguous United States and Hawaii [[1], [2], [3]]. A genome assembly is available (GenBank accession GCA_928085145.1), although a description of it has not yet been published. Accurate annotation of this species’ gene repertoire will largely depend on adequate extrinsic information, including messenger RNA sequence data spanning developmental stadia, sexes and tissue types. In addition, digital transcriptomic data provide important information as to whether a gene is differentially expressed and, if so, in what contexts.

Some transcriptomic resources for this insect are available in the literature, including a midgut-specific dataset [4] and whole-insect male and female adult samples [5]. The resources contributed here expand on those by characterizing whole-insect samples for 2nd and 4th nymphal instars. To ensure comparability of these juvenile data with adult gene expression measurements, data were also generated for male and female adults using identical library preparation protocols and by sequencing on a single instrument, minimizing the potential influence of confounding variables.

## 3 Data Description

Unassembled, quality-trimmed Illumina RNA-Seq read data and a transcriptome assembly are available from NCBI's SRA and TSA divisions, respectively, under BioProject accession number PRJNA1151593. These data are intuitively organized by the NCBI as a matter of routine and are accessible via ordinary web hyperlinks or by the NCBI's ‘SRA Toolkit’ software (see https://github.com/ncbi/sra-tools). Gene expression and annotation information, based on a limited analysis of the assembled transcriptome data reported here, are provided in the supplementary file, “SGSB_SuppFile.exprAnnot.xlsx”, available from the Open Science Framework at https://doi.org/10.17605/OSF.IO/HPQDR.

## 4 Experimental Design, Materials and Methods

**Fig. 1:**
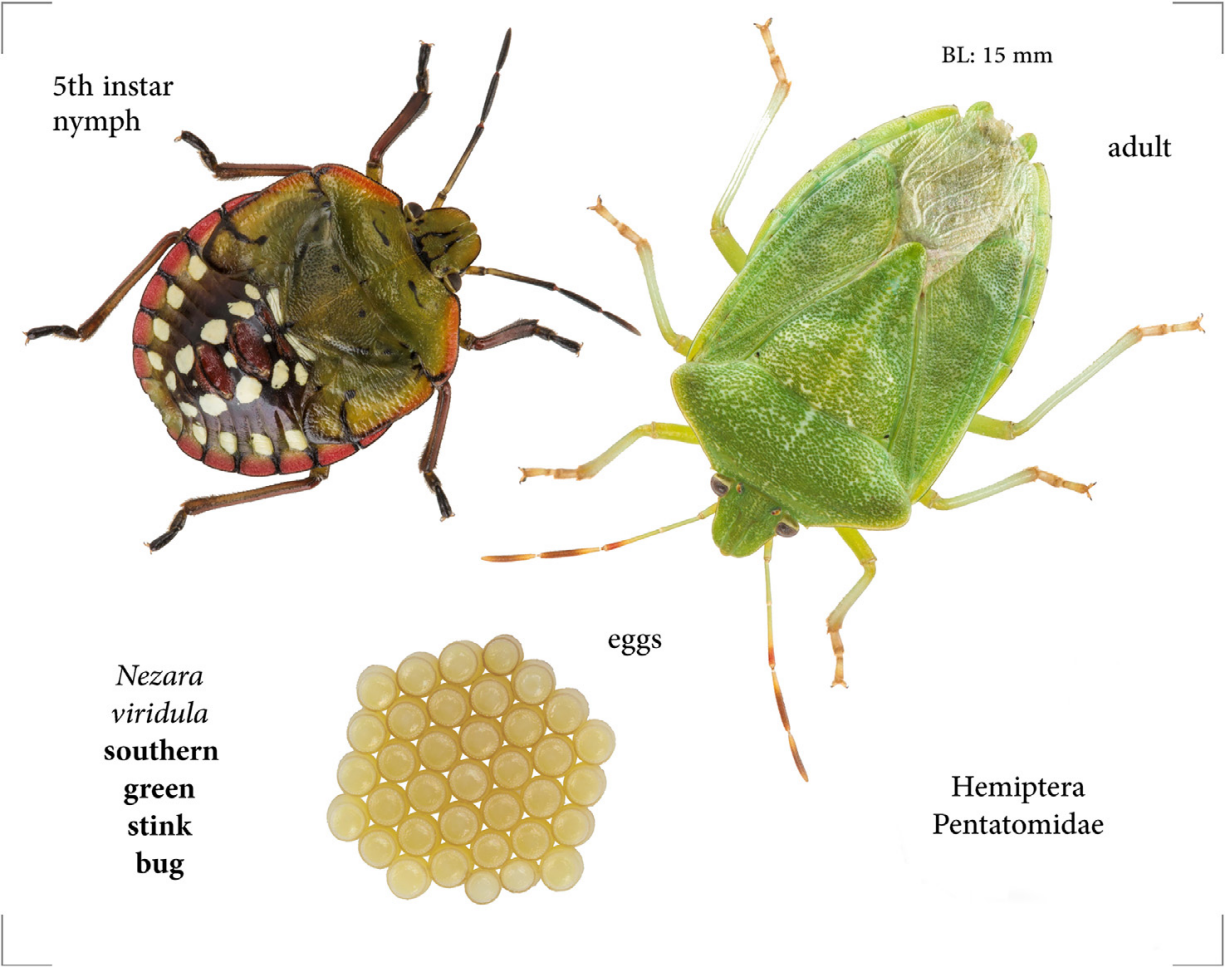
*Nezara viridula* adult, nymph and egg mass. Insect images copyright Alex Surcică and used herein with permission.

Southern green stink bugs (visually depicted in Fig. 1) were obtained from a colony maintained at the USDA-ARS Invasive Insect Biocontrol and Behavior Laboratory in Beltsville, Maryland, USA. This colony originated from field collections near Tifton, Georgia, USA, and had been continuously maintained in culture at Beltsville with no additional introductions of field-collected insects up to the point of sampling. Insects were reared in ventilated plastic cylinders (21 × 21 cm OD) on a diet consisting of organic green beans, buckwheat seeds and shelled sunflower (2∶1, w/w), and distilled water supplied in cotton-stopped shell vials. Eggs were collected on a twice-weekly basis and were hatched in plastic Petri dishes with a water vial; after molting to second instars, nymphs were then transferred to larger rearing cages for progression through the remaining four nymphal instars. Male and female adults were separated at one-to-two days post final molt and maintained in separate containers thereafter. Insects were maintained in Percival growth chambers (Percival Scientific, Perry, Iowa, USA) at 25 °C and 50±20 % relative humidity, under a 16L:8D photoperiod.

Three biological replicates apiece were prepared for each of 2nd and 4th nymphal instars, and approximately seven-day-old unmated male and female adults. Biological replicates for 2nd instar nymph samples consisted of ten pooled individuals apiece; replicates for 4th instar nymphs, as well as both male and female adults, each consisted of five pooled individuals. Whole insects were placed in 2 mL matrix A lysing tubes and pulverized using MP BioMedicals’ (Solon, Ohio, USA) Fastprep 24 for 60 s in 300 µl lysis buffer. RNA was extracted utilizing the RNAqueousTM Total RNA Isolation kit (ThermoFisher, Waltham, Massachusetts, USA) protocol and further purified using Turbo DNAse (ThermoFisher, Waltham, Massachusetts, USA) to remove any DNA contamination. RNA concentration was determined using Promega's (Madison, Wisconsin, USA) Quantas fluorometer.

Samples were aliquoted and sent to the University of Georgia Genomics Facility (Athens, Georgia, USA) for Illumina PE150 RNA-Seq sequencing. Quality-based read trimming was performed by the vendor and total sequencing volumes achieved are presented in Table 1. Reads from all 12 samples were combined and *in silico* normalized using utilities provided with the Trinity RNA-Seq assembler (v2.15.1; [6]). Normalized reads were then aligned to an as-yet unpublished *Nezara viridula* genome assembly, available under GenBank accession identifier GCA_928085145.1—using the STAR alignment program (v2.7.9a_2021–06–25; [7]). Short read alignment results were merged and used to produce a genome-guided transcriptome assembly with Trinity. The assembly consisted of 465,877,026 base pairs assembled into 599,558 transcripts, of which 599,436 were unique.

**Table 1:** Raw sample-specific sequencing volumes. A target length of 150 bp was used for each paired read.

	2nd Instar Nymphs		
	*biorep 1*	*biorep 2*	*biorep 3*
read pairs	51,563,878	34,363,285	68,282,804
bases	14,988,944,943	9,722,040,871	19,232,202,762


	4th Instar Nymphs		
	*biorep 1*	*biorep 2*	*biorep 3*
read pairs	47,451,689	17,595,829	47,062,678
bases	13,613,956,620	5,018,687,321	13,211,987,733


	Male Adults		
	*biorep 1*	*biorep 2*	*biorep 3*
read pairs	29,323,945	41,897,168	50,247,275
bases	8,297,979,878	11,817,644,886	14,097,544,270


	Female Adults		
	*biorep 1*	*biorep 2*	*biorep 3*
read pairs	39,070,563	39,040,391	28,392,487
bases	11,109,331,738	10,775,738,665	7,914,408,665

Assembled transcripts were compared with the 1 July 2024 NCBI NR protein database using DIAMOND (v2.0.4; [8]), operating in its BLASTx-like mode with default parameters. In addition, a longest open reading frame (ORF) was found for each mRNA sequence after translation in all six frames using the transeq program from EMBOSS (v6.6.0.0; [9]). Longest ORFs were then compared with the Pfam database (accessed 12 September 2023; [10]) using HMMer (v3.3.4; [11]) with default parameters. Associated Gene Ontology (GO) terms for protein family matches were identified using the pfam2go table (v2024-01-17; [12]). A total of 137,371 transcripts (22.9 % of total) exhibited one or more hits to reference protein sequences, with the remainder corresponding to transcripts not yet annotated in reference databases, transcripts whose sequences have diverged too extensively for homology to be detected by the alignment algorithm, misassembled transcripts or yet other possibilities. Protein family (Pfam) hits for inferred translation products were observed for 53,557 transcripts (8.9 % of total), and of these, 15, 615 (29.2 %) could be linked with Gene Ontology information. These sequence annotations, as well as transcription expression measurements (see below), are provided on the worksheet labeled “all_isoforms_tct-level_TPM” of the supplementary file, “SGSB_SuppFile.exprAnnot.xlsx” (available for download from the Open Science Framework at https://doi.org/10.17605/OSF.IO/HPQDR) .

**Fig. 2:**
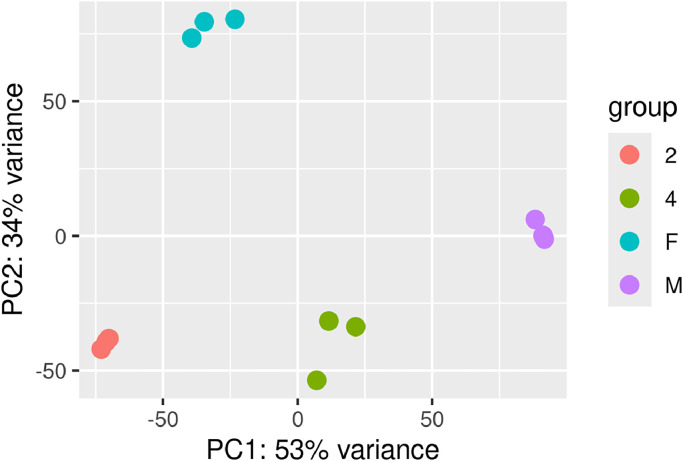
Principal component analysis of gene expression among all 12 RNA-Seq samples. Legend labels correspond to the following sample types: 2 ∼ 2nd nymphal instars, 4 ∼ 4th nymphal instars, F ∼ female adults, and M ∼ male adults.

DESeq2 (v1.36.0; [13]), used together with the salmon mapping tool (v1.9.0; [14]), identified differentially expressed genes (DEGs) with statistical significance in all six possible pairwise comparisons among the four sample types: 2nd nymphal instar vs 4th nymphal instar, 2nd nymphal instar vs male adult, 2nd nymphal instar vs female adult, 4th nymphal instar vs male adult, 4th nymphal instar vs female adult, and female adult vs male adult. An aggregated comparison in which 2nd and 4th instar nymphs were pooled and compared against a similarly pooled set of adult male and female reads was also performed. A gene was designated as differentially expressed if it exhibited a false discovery rate of 0.05 or less and at least a doubling of mean abundance between the sample types being compared. As a quality control check, a principal component analysis was performed on all 12 RNA-Seq samples using the vst and plotPCA tools from the DESeq2 package—results demonstrate that sample types group unambiguously on the basis of gene expression variance (see Fig. 2). To obtain both gene- and transcript-level expression estimates, the expression estimation method implemented in RSEM (v1.3.3; [15]), based upon read alignment results produced by bowtie 2 (v2.4.5; [16]), was invoked by the Trinity software package's align_and_estimate_abundance utility. Expression measurements were conveyed using the Transcripts per Million unit (TPM; [17]) and are provided in the aforementioned supplementary file.

**Fig. 3:**
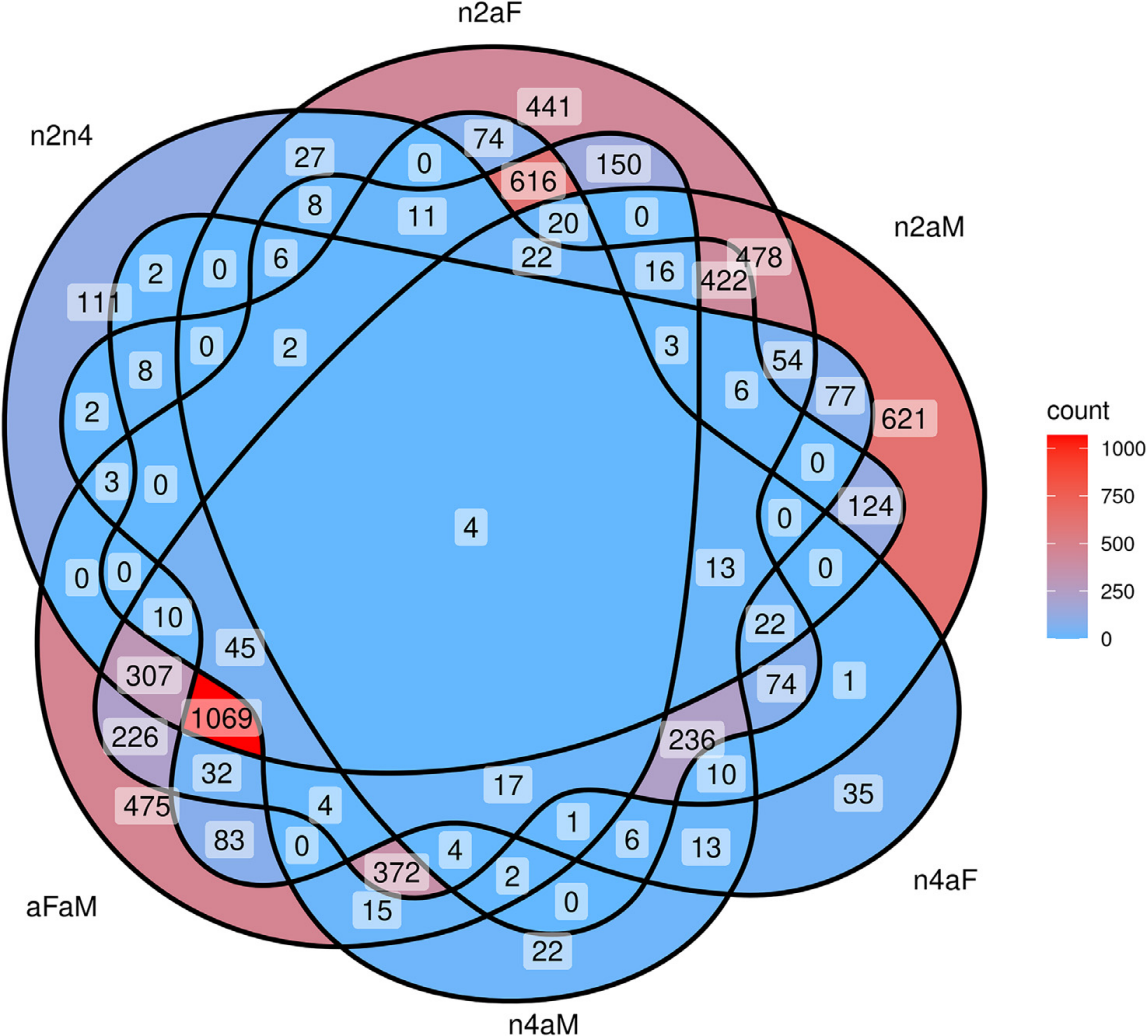
Venn diagram depicting counts of shared (or unique) differentially expressed genes (DEGs) among the six basic sample comparisons considered in this study. Note that whether a DEG was up- or down-regulated was not considered for the amounts reported here—that is, a DEG up-regulated in one context and down-regulated in another was counted as a single DEG. Comparison labels indicate the following: n2n4 ∼ 2nd nymphal instar vs 4th nymphal instar, n2aM ∼ 2nd nymphal instar vs male adult, n2aF ∼ 2nd nymphal instar vs female adult, n4aM ∼ 4th nymphal instar vs male adult, n4aF ∼ 4th nymphal instar vs female adult, and aFaM ∼ female adult vs male adult.

Differential gene expression analysis yielded the following overall DEG counts: 2nd nymphal instar vs 4th nymphal instar = 2243 DEGs (426 up, 1817 down); 2nd nymphal instar vs male adult = 4289 DEGs (1250 up, 3039 down); 2nd nymphal instar vs female adult = 2735 DEGs (985 up, 1750 down); 4th nymphal instar vs male adult = 932 DEGs (312 up, 620 down); 4th nymphal instar vs female adult = 2423 DEGs (1430 up, 993 down) ; female adult vs male adult = 3523 DEGs (1257 up, 2266 down) ; and pooled nymphs vs pooled adults = 2494 DEGs (1569 up, 925 down). A comprehensive listing of all DEGs, including fold change information (expressed in binary logarithmic space) and adjusted P-value data, is presented on the worksheet labeled “contrasts_with_gene-level_TPM” of the supplemental file. A Venn diagram summarizing counts of DEGs shared among the six basic, non-pooled sampled comparisons, prepared using the ggVennDiagram R package [18], is presented in Fig. 3.

A total of 21,380 transcripts expressed from the nonredundant set of DEGs described above were identified. A total of 15,397 (72.0 %) of these had a hit with the NCBI NR database, 8372 (39.2 %) exhibited one or more hits to reference Pfam protein domain families and 2811 of these (33.6 %) had one or more associated GO terms. Annotation information is provided on the supplemental file's “DEG-associated_isoforms” sheet.

## Limitations

*None*.

## Ethics Statement

The authors have read and followed the ethical requirements for publication in *Data in Brief* and confirm that the current work does not involve human subjects, animal experiments, or any data collected from social media platforms.

## Credit Author Statement

**Michael E. Sparks:** Conceptualization, Methodology, Formal analysis, Data Curation, Writing - Original Draft. **Daniel Kuhar:** Resources, Investigation. **Donald C. Weber:** Conceptualization, Resources, Writing - Original Draft. **Dawn E. Gundersen-Rindal:** Conceptualization, Supervision, Writing - Original Draft.

## Data Availability

Open Science FrameworkN. viridula gene expression and annotation data, supplementary information (Original data). NCBINezara viridula transcriptome, USDA-ARS (Original data).
